# Supplementary material for: The Polymers of Diethynylarenes—Is Selective Polymerization at One Acetylene Bond Possible? A Review
Source: Polymers (Basel). 2023 Feb 22;15(5):1105. doi: 10.3390/polym15051105 (PMC10006943; doi:10.3390/polym15051105)
Supplement: Supplementary file 1 [file polymers-15-01105-s001.zip › polymers-2131817-Table S1.pdf]

Table S1. Methods of synthesis of DEB polymers and the type of their intramolecular structure.[a]

Synthesis Conditions or the Catalyst Used	Declarated Type of Structure	Original Authors Notes	References in Main Text
		**Poly-p-DEB**	
Solid-phase polymerization under pressure in combination with shear deformation	Crosslinked polymer	-	[40]<br>40. Kapustyan, V.M.; et al. *Dokl. Akad. Nauk SSSR* 1968, *179*, 627–628.
UV-irradiation of crystals	Branched and crosslinked polymer	-	[41-46]<br>41. Broude, V.L.; et al. *Khim. Vys. Energ.* **1968**, *2*, 165–171.<br>42. Gordon, D.A.; et al. *J. Photochem. Photobiol., A* 1995, *86*, 253–257, doi:10.1016/1010-6030(94)03938-Q.<br>43. Gordon, D.A.; et al. *J Low Temp Phys* 2005, *139*, 675–681, doi:10.1007/s10909-005-5479-6.<br>44. Gordon, D.A.; et al. *Low Temp. Phys.* 2009, *35*, 269–274, doi:10.1063/1.3114591.<br>45. Gordon, D.A.; et al. *J. Polym. Sci. B Polym. Phys.* 1994, *32*, 2405–2411, doi:10.1002/polb.1994.090321411.<br>46. Gordon, D.A et al. *Russ. J. Phys. Chem.* 2014, *88*, 2129–2136, doi:10.1134/S0036024414120085.
γ-irradiation of crystals	Branched and crosslinked polymer	-	[41,44-49]<br>41. Broude, V.L.; et al. *Khim. Vys. Energ.* **1968**, *2*, 165–171.<br>44. Gordon, D.A.; et al. *J. Photochem. Photobiol., A* **1995**, *86*, 253–257, doi:10.1016/1010-6030(94)03938-Q.<br>45. Gordon, D.A.; et al. *J Low Temp Phys* **2005**, *139*, 675–681, doi:10.1007/s10909-005-5479-6.<br>46. Gordon, D.A.; et al. *Low Temp. Phys.* **2009**, *35*, 269–274, doi:10.1063/1.3114591.<br>47. Gordon, D.A.; et al. *J. Polym. Sci. B Polym. Phys.* **1994**, *32*, 2405–2411, doi:10.1002/polb.1994.090321411.<br>48. Gordon, D.A.; et al. *Russ. J. Phys. Chem.* **2014**, *88*, 2129–2136, doi:10.1134/S0036024414120085.<br>49. Gordon, D.A et al. *High Energy Chem* 2014, *48*, 5–10, doi:10.1134/S0018143914010068.
γ-irradiation of crystals	Soluble product	Composition: dimers, trimers, unreacted monomers	[27]<br>27. Li, Q.; et al. *Polym. Degrad. Stab.* 2015, *114*, 81–88, doi:10.1016/j.polymdegradstab.2015.01.018.
γ-irradiation of a glassy solution of p-DEB in DMF	Branched polymer	-	[48,49]<br>48. Gordon, D.A.; et al. *Russ. J. Phys. Chem.* 2014, *88*, 2129–2136, doi:10.1134/S0036024414120085.<br>49. Gordon, D.A.; et al. *High Energy Chem* 2014, *48*, 5–10, doi:10.1134/S0018143914010068.
Polymerization with molecular chlorine at low temperature	Branched polymer	-	[51-53]<br>51. Kozlovskii, A.A.; et al. *Russ. J. Phys. Chem.* 2010, *84*, 1439–1444, doi:10.1134/S0036024410080285.<br>52. Gordon, D.A.; et al. *Low Temp. Phys.* 2013, *39*, 565–570, doi:10.1063/1.4811266.<br>53. Gordon, D.A.; et al. *Russ. J. Phys. Chem. B* 2016, *10*, 699–706, doi:10.1134/S1990793116040229.
Vacuum deposition on Cu(111) substrates and annealing at 175–350°C	Covalent aggregates and meshes	-	[54]<br>54. Eichhorn, J.; et al. *Chem. Commun.* 2013, *49*, 2900, doi:10.1039/c3cc40444g.
Thermal frontal polymerization of evaporating p-DEB	Carbon material with a graphite structure	Presence of microspheres with a narrow (100–200 nm) pore size distribution	[55]<br>55. Volkova, N.N.; et al. *IOP Conf. Ser.: Mater. Sci. Eng.* 2019, *693*, 012027, doi:10.1088/1757-899X/693/1/012027.

Thermal melt polymerization	Crosslinked polymer	-	[56,61]<br>56. Cherkashin, M.I.; et al. *Russ Chem Bull* 1967, *16*, 2335–2337, doi:10.1007/BF00911839.<br>61. Berlin, A.A.; et al., USSR Patent SU-191800-A, filed 22.09.1965, and issued 1967.
Thermal melt curing	-	The activation energies of the curing reaction o-, m-, p-DEB are compared	[62]<br>62. Sastri, S.B.; et al. *Macromolecules* 1993, *26*, 6171–6174, doi:10.1021/ma00075a005.
Reaction with iodine in the melt	Soluble product	Complex composition of the branched copolymer	[63]<br>63. Grigoryan, S.G.; et al. *Polym. Sci. U.S.S.R.* 1989, *31*, 649–654, doi:10.1016/0032-3950(89)90246-3.
Boiling a solution of p-DEB in DMF (Bp=153°C)	Branched polymer	-	[64]<br>64. Tseng, W.-C.; et al. *Polym. Degrad. Stab.* 2009, *94*, 2149–2156, doi:10.1016/j.polymdegradstab.2009.09.008.
The thermal reaction. The technique is not complete	The gel was transferred to a crosslinked polymer	A composite with fir powder was obtained	[65]<br>65. Wang, Y.-G.; et al. *Chinese Journal of Applied Chemistry* 2010, *27*, 418–423, doi:10.3724/SP.J.1095.2010.90354.
UV-light or γ-^{60}Co irradiations of p-DEB solutions in ethanol	Lack of polymerization	-	[41,43]<br>41. Broude, V.L.; et al. *Khim. Vys. Energ.* 1968, *2*, 165–171.<br>43. Gordon, D.A.; et al.*J Low Temp Phys* 2005, *139*, 675–681, doi:10.1007/s10909-005-5479-6.
$(i\text{-}C_4H_9)_3Al—TiCl_4$ (Al/Ti=0.5 mol)	Crosslinked polymer	-	[68,69]<br>68. Ermakova, V.D.; et al. *Russ Chem Bull* 1972, *21*, 1139–1141, doi:10.1007/BF00853790.<br>69. Yermakova, V.D.; et al. *Polym. Sci. U.S.S.R.* 1974, *16*, 752–761, doi:10.1016/0032-3950(74)90391-8.
$(i\text{-}C_4H_9)_3Al—TiCl_4$ (Al/Ti=0.5 mol)	Crosslinked polymer	The structure contains phenylene fragments	[70,71]<br>70. Korshak, V.V.; et al. *Vysokomol. Soedin., Ser. B* 1972, *14*, 886–888.<br>71. Sergeyev, V.A et al. *Appl. Polym. Symp.* 1975, *26*, 237–248.
$n\text{-}Bu_4Ti/(Et_3Al)_4$	Soluble polymer	Declaring a linear structure	[135]<br>135. Ai, H.; Koizumi, et al, US Patent US-4667006-A, 1987.
$[(C_2H_5O)_3P]_4{\cdot}CoI$	Crosslinked polymer	The structure contains phenylene fragments	[66,67,74]<br>66. Korshak, V.V.; et al. US Patent US-3705131-A, filed 27.11.1970, and issued 1972.<br>67. Korshak, V.V et al. *Dokl. Akad. Nauk SSSR* 1971, *201*, 112.<br>74. Korshak, V.V et al. *Vysokomol. Soedin., Ser. B* 1971, *13*, 973.
$[(C_2H_5O)_3P]_4{\cdot}CoBr$	Branched polymer with phenylene fragments	The mechanism of polymerization is proposed	[75]<br>75. Korshak, V.V.; et al. *Polym. Sci. U.S.S.R.* 1973, *15*, 28–36, doi:10.1016/0032-3950(73)90262-1.
$[(RO)_3P]_n{\cdot}CoHal$, (R = $Alk_{C\leq6}$, Hal = Cl, Br, I)	Branched polymer	The structure contains phenylene fragments	[76-79]<br>76. Korshak, V.V.; et al. *Polym. Sci. U.S.S.R.* 1973, *15*, 2465–2470, doi: 10.1016/0032-3950(73)90002-6.<br>77. Korshak, V.V. *Pure Appl. Chem.* 1974, *39*, 65–80, doi: 10.1351/pac197439010065.<br>78. Korshak, V.V. *Polym. Sci. U.S.S.R.* 1974, *16*, 1066–1087, doi:10.1016/0032-3950(74)90328-1.<br>79. Sergeev, V.A.; et al. *Russ. Chem. Rev.* 1979, *48*, 79–93, doi: 10.1070/RC1979v048n01ABEH002304.
$Ni(Acac)_2{\cdot}Ph_3P$, where acac: acetylacetonate	Branched polymer	The structure contains phe-	[80-83]<br>80. Cessna, L.C.Jr. US Patent US-3882073-A, filed

		phenylene fragments	18.12.1972, and issued 1975.<br>81. Cessna, L.C. US Patent US-4026859-A, filed 03.06.1974, and issued 1977.<br>82. Jabloner, H. US Patent US-4070333-A, filed 02.01.1974, and issued 1978.<br>83. Jabloner, H. US Patent US-4097460-A, filed 23.07.1971, and issued 1978.
$Ni(Acac)_2 \cdot Ph_3P$	Linear polymer	-	[30]<br>30. Sergeyev, V.A.; et al. *Polym. Sci. U.S.S.R.* 1986, *28*, 2638–2641, doi:10.1016/0032-3950(86)90298-4.
$Ni(Acac)_2 \cdot Ph_3P$	Branched polymer	-	[84-88]<br>84. Kirilenko, Yu.K.; et al. USSR Patent SU-771207-A, filed 17.10.1978, and issued 1980.<br>85. Kirilenko, Yu.K.; et al. USSR Patent SU-833994-A, filed 25.10.1979, and issued 1981.<br>86. Kirilenko, Yu.K.; et al. *Polym. Sci. U.S.S.R.* 1984, *26*, 2122–2127, doi:10.1016/0032-3950(84)90455-6.<br>87. Cherkashin, M.I.; et al. *Vysokomol. Soedin., Ser. B* 1985, *27*, 9–11.<br>88. Kirilenko, Yu.K.; et al. *Fibre Chem* 1985, *16*, 429–430, doi:10.1007/BF00546265.
$Ni(C_5H_7O_2)_2 \cdot Ph_3P$	Branched polymer	The structure contains phenylene fragments	[64]<br>64. Tseng, W.-C.; et al. *Polym. Degrad. Stab.* 2009, *94*, 2149–2156, doi:10.1016/j.polymdegradstab.2009.09.008.
$Ni(C_5H_7O_2)_2 \cdot Ph_3P$	Branched polymer	The structure contains phenylene fragments	[89]<br>89. Oishi, S.S.; et al. *Polímeros* 2014, *24*, 541–546, doi:10.1590/0104-1428.1623.
Rh(cod)acac and Rh(nbd)acac (cod: cycloocta-1,5-diene; nbd: norborna-2,5-diene; acac: acetylacetone)	Crosslinked polymer	Cross-linking with arylene linkers. Polymers contain –C≡C–H groups	[14,15]<br>14. Hanková, V.; et al. *Macromol. Rapid Commun.* 2012, *33*, 158–163, doi:10.1002/marc.201100599.<br>15. Slováková, E.; et al. *Macromol. Chem. Phys.* 2014, *215*, 1855–1869, doi:10.1002/macp.201400198.
$[Rh(nbd)Cl]_2/Et_3N$	Crosslinked polymer	Cross-linking with arylene linkers. Polymers contain –C≡C–H groups	[15]<br>15. Slováková, E.; et al. Macromol. Chem. Phys. 2014, 215, 1855–1869, doi:10.1002/macp.201400198.
Rh(nbd)acac	Crosslinked polymer	Cross-linking with arylene linkers. Evaluation of the catalytic activity of acidic hydrogen ≡C–H	[90,91]<br>90. Sekerová, L.; et al. Tetrahedron 2019, 75, 2877–2882, doi:10.1016/j.tet.2019.04.013.<br>91. Sekerová, L.; et al. Chem. Eur. J. 2018, 24, 14742–14749, doi:10.1002/chem.201802432
Rh-based catalyst	-	Review	[9]<br>9. Sedláček, J.; et al. *Polym. Rev.* **2017**, *57*, 31–51, doi: 10.1080/15583724.2016.1144207.
$Ni(PPh_3)_2(C{\equiv}CC_6H_4C{\equiv}CH)_2$	The structure of a linear trans-polyene is declared	Lack of structure studies. Humidity sensors. Doping by $HClO_4$	[92-94]<br>92. Casalbore-Miceli, G. *Solid State Ionics* **1997**, *100*, 217–224, doi: 10.1016/S0167-2738(97)00349-4.<br>93. Yang, M.; et al. *Chin. Chem. Lett.* **1998**, *9*, 223–225.<br>94. Zhan, X.; et al. *Synth. Met.* **1998**, *94*, 249–253, doi: 10.1016/S0379-6779(98)00007-1.
$Ni(PPh_3)_2(C{\equiv}CC_6H_4C{\equiv}CH)_2$	Branched	Humidity	[95]

	trans-polyene	sensors	95. Yang, M.; et al. *J. Appl. Polym. Sci.* **1999**, *74*, 2010–2015, doi: 10.1002/(SICI)1097-4628(19991121)74:8<2010::AID-APP16>3.0.CO;2-1.
$Ni(PPh_3)_2(C{\equiv}CC_6H_4C{\equiv}CH)_2$	The structure of a linear trans-polyene is declared	Optical, photoluminescent and electroluminescent properties	[96]<br>96. Yu, G.; et al. *Thin Solid Films* **2000**, *363*, 126–129, doi: 10.1016/S0040-6090(99)01016-0.
$Ni(PPh_3)_2(C{\equiv}CC_6H_4C{\equiv}CH)_2$	Branched trans-polyene	Photoluminescence, electroluminescence and conductivity of doped by $HClO_4$, H_2SO_4, HBF_4, I_2, $FeCl_3$, CH_3COOH polymers	[97]<br>97. Zhan, X.; et al. *Adv. Mater.* **2000**, *12*, 51–53, doi: 10.1002/(SICI)1521-4095(200001)12:1<51::AID-ADMA51>3.0.CO;2-J.
$Ni(PPh_3)_2(C{\equiv}CC_6H_4C{\equiv}CH)_2$	Branched trans-polyene	-	[98]<br>98. Yang, M.; et al. *Chin. J. Polym. Sci.* **2001**, *19*, 303–309.
$Ni(PPh_3)_2(C{\equiv}CC_6H_4C{\equiv}CH)_2$	Linear trans-polyene on the scheme	Lack of structure studies.	[100]<br>100. Zhan, X.; et al. *J. Mol. Catal. A: Chem.* **2001**, *169*, 27–31, doi:10.1016/S1381-1169(00)00559-8.
$Ni(PPh_3)_2(C{\equiv}CC_6H_4C{\equiv}CH)_2$	Branched trans-polyene	Doping by $FeCl_3$. Conductivity	[102]<br>102. Zhan, X.; et al. *Eur. Polym. J.* **2002**, *38*, 2057–2061, doi: 10.1016/S0014-3057(02)00091-5.
$Ni(PPh_3)_2(C{\equiv}CC_6H_4C{\equiv}CH)_2$	A linear trans-polyene is declared, but the synthesis is carried out in accordance with [95], where the structure of a branched trans-polyene is given	Humidity sensors	[103]<br>103. Li, Y.; et al. *Sens. Actuators, B* **2007**, *122*, 560–563, doi: 10.1016/j.snb.2006.06.031.
$Ni(PPh_3)_2(C{\equiv}CCH_2OCOCH_3)_2$,<br>$Ni(PBu_3)_2(C{\equiv}CC_6H_4C{\equiv}CH)_2$,<br>$Ni(PBu_3)_2(C{\equiv}CCH_2OCOCH_3)_2$	Branched polymer	-	[99]<br>99. Zhan, X.; et al. *Macromol. Rapid Commun.* **2001**, *22*, 530–534, doi: 10.1002/1521-3927(20010401)22:7<530::AID-MARC530>3.0.CO;2-N.
$NiL_2(C{\equiv}CR)_2$; $NiL_2(C{\equiv}CR)Cl$ and $Ni(PBu_3)_2Cl_2$, where L_2 = $(PPh_3)_2$, $(PBu_3)_2$ and $Ph_2PCH_2CH_2PPh_2$; R = p-$C_6H_4C{\equiv}CH$, C_6H_5, H, CH_2OH and CH_2OCOCH_3	Soluble polymer. Structure is not shown.	Lack of structure studies.	[100]<br>100. Zhan, X.; et al. *J. Mol. Catal. A: Chem.* **2001**, *169*, 27–31, doi: 10.1016/S1381-1169(00)00559-8.
(π-C_5H_5)$LNi(C{\equiv}CR)$ (where L = PPh_3, PBu_3; R = p-$C_6H_4C{\equiv}CH$, C_6H_5, H)	Branched polymer	-	[104]<br>104. Zhan, X.; et al. *Macromol. Rapid Commun.* **2000**, *21*, 1263–1266, doi: 10.1002/1521-3927(20001101)21:17<1263::AID-MARC1263>3.0.CO;2-X.
(π-C_5H_5)$(PPh_3)Ni(C{\equiv}CC_6H_4C{\equiv}CH)$	Branched trans-polyene	-	[105]<br>105. Zhan, X.; et al. *Eur. Polym. J.* **2001**, *37*, 1649–1654, doi: 10.1016/S0014-3057(01)00016-7.
(π-C_5H_5)$(PPh_3)Ni(C{\equiv}CC_6H_4C{\equiv}CH)$	Branched trans-polyene	Doping by I_2. Conductivity.	[106]<br>106. Zhan, X.; et al. *Eur. Polym. J.* **2002**, *38*,

			2349–2353, doi: 10.1016/S0014-3057(02)00144-1.
Unknown Ni catalyst	Branched trans-polyene structure is declared	Lack of structure studies. Comparison of the curing processes of soluble poly-p-DEB and poly-m-DEB	[107]<br>107. Ding, X.; et al. *J. East China Univ. Sci. Technol.* **2001**, *27*, 161–164.
Unknown Ni catalyst	Branched trans-polyene structure is declared	Lack of structure studies. The study of the rheology of prepolymers synthesized in [100]	[108]<br>108. Ding, X.; et al. *J. Funct. Polym.* **2001**, *14*, 105–108.
(π-C_5H_5)(PPh_3)NiCl	Soluble polymer. Structure not specified	Lack of structure studies.	[100]<br>100. Zhan, X.; et al. *J. Mol. Catal. A: Chem.* **2001**, *169*, 27–31, doi: 10.1016/S1381-1169(00)00559-8.
$Ni(PPh_3)_2Cl_2$	Catalyst not active		[100]<br>100. Zhan, X.; et al. *J. Mol. Catal. A: Chem.* **2001**, *169*, 27–31, doi: 10.1016/S1381-1169(00)00559-8.
$Pd(PPh_3)_2(C{\equiv}CCH_2OH)_2$	Branched trans-polyene	-	[109]<br>109. Zhan, X.; et al. *J. Mol. Catal. A: Chem.* **2002**, *184*, 139–145, doi:10.1016/S1381-1169(02)00006-7.
$Pd(PPh_3)_2(C{\equiv}CC_6H_4C{\equiv}CH)_2$	Branched trans-polyene	-	[99]<br>96. Zhan, X.; et al. *Macromol. Rapid Commun.* **2001**, *22*, 530–534, doi:10.1002/1521-3927(20010401)22:7<530::AID-MARC530>3.0.CO;2-N.
$Pd(PPh_3)_2(C{\equiv}CC_6H_4C{\equiv}CH)_2$	Branched trans-polyene	Third-order nonlinear optical properties	[110]<br>110. Zhan, X.; et al. *Macromol. Rapid Commun.* **2001**, *22*, 358–362, doi:10.1002/1521-3927(20010301)22:53.3.CO;2-O.
$Pd(PBu_3)_2(C{\equiv}CC_6H_4C{\equiv}CH)_2$	Catalyst not active		[99]<br>96. Zhan, X.; et al. *Macromol. Rapid Commun.* **2001**, *22*, 530–534, doi:10.1002/1521-3927(20010401)22:7<530::AID-MARC530>3.0.CO;2-N.
$Pd(PPh_3)_2(C{\equiv}CR)_2$ где R = p-$C_6H_4C{\equiv}CH$, C_6H_5, H, CH_2OH, CH_2OCOCH_3, $CH_2OCOC_6H_5$, $CH_2OCOC_6H_4OH$-o	Probably, branched trans-polyene	Lack of structure studies.	[111]<br>111. Zhan, X.; et al. *J. Mol. Catal. A: Chem.* **2001**, *169*, 63–66, doi:10.1016/S1381-1169(01)00037-1.
$Pd(PBu_3)_2$ $(C{\equiv}CCH_2OH)_2$<br>$Pd(PBu_3)_2$ $(C{\equiv}CC_6H_4C{\equiv}CH)_2$	Catalyst not active		[111]<br>111. Zhan, X.; et al. *J. Mol. Catal. A: Chem.* **2001**, *169*, 63–66, doi:10.1016/S1381-1169(01)00037-1
$Co(PPh_3)_2(C{\equiv}CC_6H_5)_2$<br>$Ni(PPh_3)_2(C{\equiv}CC_6H_5)_2$<br>Pd(PPh3)2($C{\equiv}CC_6H_5)_2$<br>$Co(PBu_3)_2Cl_2$<br>$Ni(PBu_3)_2Cl_2$<br>$Ni(PPh_3)_2Cl_2$<br>$Pd(PPh_3)_2Cl_2$	Branched trans-polyene		109. Zhan, X.; et al *J. Mol. Catal. A: Chem.* **2002**, *184*, 139–145, doi:10.1016/S1381-1169(02)00006-7.
$Pt(PPh_3)_2(C{\equiv}CC_6H_5)_2$<br>$Pd(PBu_3)_2Cl_2$<br>$Pt(PBu_3)_2Cl_2$<br>$Co(PPh_3)_2Cl_2$	Catalyst not active		109. Zhan, X.; et al *J. Mol. Catal. A: Chem.* **2002**, *184*, 139–145, doi:10.1016/S1381-1169(02)00006-7.

$Pt(PPh_3)_2Cl_2$			
$Co_2(CO)_8$	Insoluble polymer	The polymer contains phenylene fragments and no –C≡C–H groups	[112]<br>112. Yuan, S.; et al. *Chem. Commun.* **2010**, *46*, 4547, doi:10.1039/c0cc00235f.
$Mo(CO)_6$+УФ	Soluble polymer	A linear polyene structure is declared. There are –C≡C–H groups	[135]<br>135. Ai, H.; Koizumi, et al, US Patent US-4667006-A, 1987
$W(CO)_6$+УФ	Soluble polymer	A linear polyene structure is declared. There are –C≡C–H groups	[135]<br>135. Ai, H.; Koizumi, et al, US Patent US-4667006-A, 1987
$[Co_2(CO)_6]_2$·Ph-C≡C-C≡C-Ph	Cross-linked polyene with pendants –Ph–C≡C–H	-	[110,111]<br>110. Misin, V.M.; et al. *ChemistrySelect* **2022**, *7*, e202103612, doi:10.1002/slct.202103612.<br>111. Glagolev, N.N.; et al. Aghveran, USSR, 1984; pp. 30–31.
Probably, $NbCl_5$	Insoluble polymer	-	[112]<br>112. Srinrivasan, R.; et al. *Polym. Bull.* **1988**, *20*, doi:10.1007/BF00255737.
WCl_6	Soluble polymer. After 30 minutes a gel is formed.	A linear structure is declared. There are –C≡C–H groups	[135]<br>135. Ai, H.; Koizumi, et al, US Patent US-4667006-A, 1987
Probably one of the catalysts TaX5-Ph4Sn, (X=Cl, Br); TaBr5, CpCo(CO)2 − hν	Insoluble polymer	-	[21]<br>21. Häußler, M.; et al. *C. R. Chim.* **2003**, *6*, 833–842, doi: 10.1016/j.crci.2003.06.003.
$TaCl_5/Ph_4Sn$	Insoluble polymer	The polymer contains phenylene fragments and –C≡C–H groups	[116]<br>116. Zukal, A.; et al. *Macromol. Chem. Phys.* **2013**, *214*, 2016–2026, doi:10.1002/macp.201300317.
WCl_6/Ph_4Sn	Polymerization is ineffective		[15]<br>15. Slováková, E.; et al. Macromol. Chem. Phys. 2014, 215, 1855–1869, doi:10.1002/macp.201400198
$MoCl_5/Ph_4Sn$	Polymerization is ineffective		[15]<br>15. Slováková, E.; et al. Macromol. Chem. Phys. 2014, 215, 1855–1869, doi:10.1002/macp.201400198
PR_3 (R = Me_2NO, Et, Ph, Bu)	Insoluble polymer	Lack of structure studies.	[119]<br>119. Gasparyan, G.Ts.; et al. *Armyanskij himicheskij zhurnal* **1988**, *41*, 716–717.
Electrically initiated anionic polymerization	Soluble polymer	There are probably branches.	[121]<br>121. Grovu-Ivanoiu, M.; et al. *Makromol. Chem.* **1985**, *186*, 2247–2254, doi: 10.1002/macp.1985.021861105.
In CH_3NH_2 medium at 260°C	Insoluble polymer	They declare the presence of phenylene fragments and the absence of –C≡C–H groups. Lack of structure studies.	[122]<br>122. Sergeev, V.A.; et al. *Vysokomol. Soedin., Ser. B* **1977**, *19*, 899–903.

n-BuLi	Linear polymer	Each polymer unit contains –C≡C–H groups	[33,124-126]<br>33. Misin, V.M.; et al. *Polymers* **2022**, *14*, 900, doi:10.3390/polym14050900.<br>124. Misin, V.M.; et al. *Vysokomol. Soedin., Ser. B* **1981**, *23*, 130–131.<br>125. Glagolev, N.N.; et al. Pat. SU-910662-A1, 1981.<br>1262. Glagolev, N.N.; et al. *Sov. J. Chem. Phys.* **1990**, *5*, 1628–1640.
n-BuLi	Linear polymer	Modification of polymers with $Co_2(CO)_8$, $B_{10}H_{14}$, CuCl	[29,31-33]<br>29. Glagolev, N.N.; et al.; Yerevan, USSR, 1984; p. 211.<br>31. Misin, V.M.; et al. *Russ J Appl Chem* **2007**, *80*, 1910–1913, doi:10.1134/S1070427207110262.<br>32. Misin, V.M.; et al. *Vestn. Kazan. Tekhnol. Univ.* **2016**, *19*, 50–59.<br>33. Misin, V.M.; et al. *Polymers* **2022**, *14*, 900, doi:10.3390/polym14050900.
n-BuLi	Linear polymer	Synthesis of skin layers by vacuum deposition of a polymer.	[127,128]<br>127. Misin, V.M.; et al.; Gomel, 1990; pp. 83–84.<br>128. Misin, V.M.; et al. *Russ J Appl Chem* **2006**, *79*, 1325–1328, doi: 10.1134/S1070427206080210.
n-BuLi	Linear polymer	Modification of industrial oligoetheracrylates	[33,130-132]<br>33. Misin, V.M.; et al. *Polymers* **2022**, *14*, 900, doi:10.3390/polym14050900.<br>130. Misin, V.M et al. In *Monomers, Oligomers, Polymers, Composites and Nanocomposites Research: Synthesis, Properties and Applications (Polymer Yearbook, Volume 23)*; Polymer Science and Technology; NOVA science publishers, 2010; Vol. 23 ISBN 978-1-60876-029-9.<br>131. Misin, V.M.; et al. *Russ J Appl Chem* **2008**, *81*, 2008–2013, doi:10.1134/S107042720811027X.<br>132. Misin, V.M.; et al. *Oxid. Commun.* **2011**, *34*, 888–899.
n-BuLi	Linear polymer	Modification of industrial carbon fibers.	[33,133,134]<br>33. Misin, V.M.; et al. *Polymers* **2022**, *14*, 900, doi:10.3390/polym14050900.<br>133. Kazakov, M.E et al. *Dokl. AN SSSR* **1986**, *287*, 1139–1141.<br>134. Cherkashin, M.I.; et al.; Aghveran, USSR, 1984; pp. 87–88.
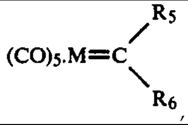 Carbenes, where M= Mo, W	They declare the linear polymer.	There are –C≡C–H groups	[135]<br>131. Ai, H.; Koizumi, et al, US Patent US-4667006-A, 1987.
Mo-Schrock carbene	Polymerization is ineffective		[15]<br>15. Slováková, E.; et al. Macromol. Chem. Phys. 2014, 215, 1855–1869, doi:10.1002/macp.201400198
$CoHal_2 \cdot 2PBu_3$ (Hal= Cl, Br)	The linear structure gradually turns into a branched one.	-	[137]<br>137. Lei, Z.; et al. *Polym J* **1998**, *30*, 879–882, doi:10.1295/polymj.30.879.
$CoHal_2 \cdot 2PBu_3$ (Hal= Cl, Br)	The structure of the polymer is not known, because no synthesis conditions given	Study of nonlinear optical properties	[134]<br>134. Yang, Q.; et al. *Chinese Phys. Lett.* **1998**, *15*, 189–191, doi:10.1088/0256-307X/15/3/013.
**Poly-o-DEB**			
$AlEt_nCl_{3-n}/TiCl_4$, $AlEt_nCl_{3-n}/Ti(acac)_3$ (where n =	Soluble or in-	Molecules con-	[145]

1,2,3), $Al(i\text{-}Bu)_3/TiCl_4$, $Al(i\text{-}Bu)_3/Ti(acac)_2$<br>$TiCl_4$, $AlEt_2Cl$, BF_3OEt_2, $AlCl_3$, $AlEtCl_2$<br>AIBN	soluble polymers depending on synthesis conditions	tain units of various structures. The number of –C≡C–H groups remaining in the polymer depends on the type of initiator.	145. Aso, C.; et al. *Macromolecular Chemistry and Physics* **1972**, *151*, 265–274, doi:10.1002/macp.1972.021510120.
Thermal polymerization	Insoluble poly(1,4-naphthylene)	-	[146 147]<br>146. John, J.A.; et al. *J. Am. Chem. Soc.* **1994**, 5011–5012, doi:10.1021/ja00090a066.<br>147. John, J.A.; et al. *Tetrahedron* **1997**, *53*, 15515–15534, doi: 10.1016/S0040-4020(97)00977-0.
Thermal polymerization	Insoluble polymer	Molecules contain units of various structures. There are –C≡C–H groups	[148]<br>148. Johnson, J.P.; et al. *J. Am. Chem. Soc.* **2003**, *125*, 14708–14709, doi:10.1021/ja035695t.
Thermal melt curing	-	The activation energies of the curing reaction o-, m-, p-DEB are compared	[62]<br>62. Sastri, S.B.; et al. *Macromolecules* 1993, *26*, 6171–6174, doi:10.1021/ma00075a005.
$MoCl_5$, WCl_6, $MoCl_5/(n\text{-}Bu)_4Sn$, $WCl_6/(n\text{-}Bu)_4Sn$, $WCl_6/EtAlCl_2$, $MoCl_5/EtAlCl_2$	Poor solubility in $CHCl_3$ and THF	Molecules contain indenylene units	[149]<br>149. Kim D.J.; et al. *Polymer (Korea)* **1994**, *18*, 297–302.
$[(nbd)RhCl]_2/Et_3N$, where nbd=2,5-norbornadiene	Insoluble polymer	Molecules contain indenylene units and –C≡C–H groups	[17]<br>17. Zhang, W.; et al. *Polymer* **2006**, *47*, 2956–2961, doi: 10.1016/j.polymer.2006.03.011.
$TaCl_5/Ph_4Sn$	Insoluble polymer	The polymer contains phenylene fragments and no –C≡C–H groups	[17]<br>17. Zhang, W.; et al. *Polymer* **2006**, *47*, 2956–2961, doi: 10.1016/j.polymer.2006.03.011.
**Poly-m-DEB**			
Probably, $[(RO)_3P]_nCoHal$, (where R = $Alk_{C\leq6}$, Hal = Cl, Br, I)	Insoluble polymer	Structure not discussed	[152]<br>152. Sergeyev, V.A.; et al. *Polymer Science U.S.S.R.* **1980**, *22*, 2130–2135, doi: 10.1016/0032-3950(80)90075-1.
WCl_6 + PhC≡C–H	Soluble polymer	A linear polyene structure is declared. There are –C≡C–H groups	[135]<br>131. Ai, H.; Koizumi, et al, US Patent US-4667006-A, 1986.
$Ni(Acac)_2 \cdot Ph_3P$	Soluble polymer	The polymer contained 10.1 $\%_{wt.}$ terminal acetylene groups	[153]<br>153. Watson, A.S. US Patent US-3993711-A, 1976.

Ni catalyst, probably $(Acac)_2Ni/2Ph_3P$	Structure of polymer not discussed	-	[154-156]<br>150. Zaldivar, R.J.; et al. *Carbon* **1991**, *29*, 1145–1153, doi: 10.1016/0008-6223(91)90032-E.<br>155. Zaldivar, R.J.; et al. *SAMPE J.* **1992**, *27*, 32.<br>156. Binegar, G.A.; et al; Space Systems Division Air Force Systems Command: Los Angeles, 1989.
Thermal melt curing	-	The activation energies of the curing reaction o-, m-, p-DEB are compared	[62]<br>62. Sastri, S.B.; et al. *Macromolecules* 1993, *26*, 6171–6174, doi:10.1021/ma00075a005.
Thermal and catalytic polymerization. Conditions not specified	Insoluble polymers	Cyclo-trimerization occurs. There are groups –C≡C–H	[162]<br>162. Cao, J.; et al. *Mater. rev.* **2001**, *08*, 64–66.
Ni-catalytic polymerization. Conditions not specified	Insoluble polymers	Cyclo-trimerization occurs. There are groups –C≡C–H	[107,108]<br>107. Ding, X.; et al. *J. East China Univ. Sci. Technol.* **2001**, *27*, 161–164.<br>108. Ding, X.; et al. *J. Funct. Polym.* **2001**, *14*, 105–108.
Probably one of the catalysts TaX5-Ph4Sn, (X=Cl, Br); TaBr5, CpCo(CO)2 − hν	Insoluble polymer		[21]<br>21. Häußler, M.; et al. *C. R. Chim.* **2003**, *6*, 833–842, doi: 10.1016/j.crci.2003.06.003
[Rh(cod)acac] and [Rh(nbd)acac] (cod: cycloocta-1,5-diene; nbd: norborna-2,5-diene; acac: acetylacetone)	Crosslinked polymer	Cross-linking with arylene linkers. Polymers contain –C≡C–H groups	[14]<br>14. Hanková, V.; et al. *Macromol. Rapid Commun.* 2012, *33*, 158–163, doi:10.1002/marc.201100599.
Rh(nbd)acac	Crosslinked polymer	Cross-linking with arylene linkers. Polymers contain –C≡C–H groups. Alkyne-azide click reaction was carried out.	[15]<br>15. Slováková, E.; et al. *Macromol. Chem. Phys.* 2014, *215*, 1855–1869, doi:10.1002/macp.201400198.
Rh-based catalyst	-	Review	[9]<br>9. Sedláček, J.; et al. *Polym. Rev.* **2017**, *57*, 31–51, doi: 10.1080/15583724.2016.1144207.
$Pd(OAc)_2/Ph_3P$/methanesulfonic acid Ultrasound was used to homogenize the reaction system. A single-stage one-pot self-encapsulating synthesis of a heterogeneous Pd catalyst was carried out.	Insoluble polymers. Simultaneous formation of cross-linked chains and encapsulation of the Pd catalyst with the formation	Assessment of catalytic activity in the Suzuki–Miyaura, Stille, allylic arylation,	[13]<br>13. Dong, Z.; et al. *Adv. Synth. Catal.* **2014**, *356*, 3401–3414, doi: 10.1002/adsc.201400520.

	of a heterogeneous Pd catalyst.	and Mizoroki–Heck reactions	
Pd(OAc)$_2$/α,α′-bis(di-t-butylphosphino)-o-xylene/ methanesulfonic acid. Ultrasound was used to homogenize the reaction system.	Insoluble polymers	Probably, there are –C≡C–H groups. Carbonization was carried out in the presence of KOH. It was recommended to use carbonizates as electrode materials in supercapacitors and as sorbents.	[164]<br>164. Grundy, M.; et al. *J. Mater. Chem. A* **2014**, *2*, 20316–20330, doi:10.1039/C4TA04038D.
Composite m-DEB/SBA-15 template cured in an oven and carbonized	Insoluble polymers	Ordered heat-resistant mesoporous coals with a very narrow pore size distribution have been synthesized.	[165]<br>165. Zhang, B.; et al. *Mater. Lett.* **2017**, *189*, 317–320, doi:10.1016/j.matlet.2016.11.008.

[a] information is taken from the primary sources indicated in the last column
