# Supplementary material for: The Polymers of Diethynylarenes—Is Selective Polymerization at One Acetylene Bond Possible? A Review
Source: Polymers (Basel). 2023 Feb 22;15(5):1105. doi: 10.3390/polym15051105 (PMC10006943; doi:10.3390/polym15051105)
Supplement: Supplementary file 1 [file polymers-15-01105-s001.zip › polymers-2131817-Table S2.pdf]

**Table S2.** Methods of synthesis of diethynylarenes polymers and the type of their intramolecular structure. [a]

| The Spacer Ar Structure | Synthesis Conditions or the Catalyst Used | Declarated Type of Structure | Original Authors Notes | References in Main Text |
|---|---|---|---|---|
| -Ph-Ph- | $[(RO)_3P]_n$·CoHal, R = $Alk_{C\leq6}$, n=1-4, Hal = Cl, Br, I | Crosslinked | Contains phenylene fragments | [64,71]<br>64. Korshak, V.V.; et al. US Patent US-3705131-A, filed 27.11.1970, and issued 1972.<br>71. Korshak, V.V., et al. *Dokl. Akad. Nauk SSSR* **1971**, *201*, 112. |
| -Ph-Ph- | i-$Bu_3Al$-$TiCl_4$ | Crosslinked | Contains phenylene fragments | [148]<br>148. Sergeyev, V.A.; et al. *Polymer Science U.S.S.R.* **1980**, *22*, 2130–2135, doi: 10.1016/0032-3950(80)90075-1. |
| -Ph-Ph- | $[Rh(cod)Cl]_2$ и [Rh(cod)im] (cod = cis,cis-cyclo- octadiene; im = imidazole) | Crosslinked | The presence of latheral groups $–C_6H_4C_6H_4C≡CH$ | [18]<br>18. Bolasco, A.; et al. *Polymer* **1992**, *33*, 3049–3054, doi:10.1016/0032-3861(92)90094-D. |
| -Ph-Ph- | $[Pd(PPh_3)_2Cl_2]$ и $[Pd(PPh_3]_2(DEDP)_2]$ | Crosslinked | The presence of latheral groups $–C_6H_4C_6H_4C≡CH$ | [18]<br>18. Bolasco, A.; et al. *Polymer* **1992**, *33*, 3049–3054, doi:10.1016/0032-3861(92)90094-D. |
| -Ph-Ph- | Thermal polymerization | Often crosslinked | Absence of groups –C≡CH | [18]<br>18. Bolasco, A.; et al. *Polymer* **1992**, *33*, 3049–3054, doi:10.1016/0032-3861(92)90094-D. |
| -Ph-Ph- | [Rh(cod)acac] and [Rh(nbd)acac] (cod: cycloocta-1,5-diene; nbd: norborna-2,5-diene; acac: acetylacetone) | Crosslinked | Cross-linking with arylene linkers. Polymers contain –C≡C–H groups | [14]<br>14. Hanková, V.; et al. *Macromol. Rapid Commun.* 2012, *33*, 158–163, doi:10.1002/marc.201100599. |
| -Ph-Ph- | $Co_2(CO)_8$ | Often crosslinked by a cyclopolymerization reaction | Absence of groups –C≡CH. The presence of 1,3,5-phenylene bridges. | [112]<br>112. Yuan, S.; et al. *Chem. Commun.* **2010**, *46*, 4547, doi:10.1039/c0cc00235f. |
| -Ph-Ph- | Annealing at 100 °C on Au(111) substrate | - | Forming of 2D networks on the Au(111) substrate | [19]<br>19. Zhou, H.; et al. *J. Am. Chem. Soc.* **2014**, *136*, 5567–5570, doi:10.1021/ja501308s. |
| -Ph-Ph- | Thermal polymerization | - | Precursor of carbon/carbon composites | [12]<br>12. Bilow, N.; et al. *SAMPE J.* **1982**, *18*, 19–24. |
| -Ph-Ph-Ph- | $[(RO)_3P]_n$·CoHal, R = $Alk_{C\leq6}$, n=1-4, Hal = Cl, Br, I | Crosslinked | Contains phenylene fragments | [64,71]<br>64. Korshak, V.V.; et al. US Patent US-3705131-A, filed 27.11.1970, and issued 1972.<br>71. Korshak, V.V., et al. *Dokl. Akad. Nauk SSSR* **1971**, *201*, 112-114. |
| Thiophenylene-2,5 | [Rh(nbd)acac] | Crosslinked | Polyconjugated chains linked by thiophene-2,5-diyl bridges. The presence of –C≡CH groups. | [20]<br>20. Bondarev, D.; et al. *Eur. Polym. J.* **2017**, *92*, 213–219, doi:10.1016/j.eurpolymj.2017.04.042. |

| | | | | |
|---|---|---|---|---|
| Thiophenylene-2,5 | $Co_2(CO)_8$ | Crosslinked | Polycyclotrimerization with the formation of nodes of 1,2,4- and 1,3,5-substituted benzenes. The almost disappearance of groups –C≡CH | [20] 20. Bondarev, D.; et al. *Eur. Polym. J.* **2017**, *92*, 213–219, doi:10.1016/j.eurpolymj.2017.04.042. |
| Naphthylene-1,4 | Photo polymerization of crystals | Soluble | Weak branching of polymer chains | [169,170] 169. Rohde, O.; et al. *Makromol. Chem.* **1978**, *179*, 1999–2011, doi:10.1002/macp.1978.021790813. 170. Rohde, O.; et al. *Makromol. Chem.* **1978**, *179*, 2013–2029, doi:10.1002/macp.1978.021790814. |
| Naphthylene-1,4 | Photo polymerization in solution | Soluble | Weak branching of polymer chains | [169,170] 169. Rohde, O.; et al. *Makromol. Chem.* **1978**, *179*, 1999–2011, doi:10.1002/macp.1978.021790813. 170. Rohde, O.; et al. *Makromol. Chem.* **1978**, *179*, 2013–2029, doi:10.1002/macp.1978.021790814. |
| 2,3-Dichloronaphthylene-1,4 | Thermal polymerization | Soluble | Weak branching of polymer chains | [[169,170] 169. Rohde, O.; et al. *Makromol. Chem.* **1978**, *179*, 1999–2011, doi:10.1002/macp.1978.021790813. 170. Rohde, O.; et al. *Makromol. Chem.* **1978**, *179*, 2013–2029, doi:10.1002/macp.1978.021790814. |
| 2,3-Dichloronaphthylene-1,4 | Photo polymerization | Soluble | Weak branching of polymer chains | [169,170] 169. Rohde, O.; et al. *Makromol. Chem.* **1978**, *179*, 1999–2011, doi:10.1002/macp.1978.021790813. 170. Rohde, O.; et al. *Makromol. Chem.* **1978**, *179*, 2013–2029, doi:10.1002/macp.1978.021790814. |
| Naphthylene-1,8 | $MoCl_5$ or $WCl_6$ | Partially soluble | Cyclization of –C≡CH groups to form a “semi-ladder” polymer with a conjugated system | [171] 171. Cho, H.N.; et al. *Polymer Bulletin* **1995**, *34*, 125–132, doi:10.1007/BF00316386. |
| Naphthylene-1,8 | $PdCl_2$ | Soluble | Cyclization of –C≡CH groups to form a “semi-ladder” polymer with a conjugated system | [171] 171. Cho, H.N.; et al. *Polymer Bulletin* **1995**, *34*, 125–132, doi:10.1007/BF00316386. |
| Naphthylene-1,8 | $MoCl_5/EtA1Cl_2$ (1:4) и $WC1_6/(n\text{-}Bu)_4Sn$ (1:4) | No information | Probably, occur cyclization of –C≡CH groups to form a “semi-ladder” polymer with a conjugated system | [171] 171. Cho, H.N.; et al. *Polymer Bulletin* **1995**, *34*, 125–132, doi:10.1007/BF00316386.. |
| Naphthylene-2,6 | $TaCl_5/Ph_4Sn$ | Insoluble | Hyperbranched cross-linked polycyclotrimers; there are few groups –C≡CH | [116] 116. Zukal, A.; et al. *Macromol. Chem. Phys.* **2013**, *214*, 2016–2026, doi:10.1002/macp.201300317. |
| Антрилен-2,6 | $TaCl_5/Ph_4Sn$ | Insoluble | Hyperbranched cross-linked polycyclotrimers; there are few groups –C≡CH | [116] 116. Zukal, A.; et al. *Macromol. Chem. Phys.* **2013**, *214*, 2016–2026, doi:10.1002/macp.201300317. |
| -Ph-O-Ph- | Thermal polymerization | - | Precursor of carbon/carbon composites | [12] 12. Bilow, N.; et al. *SAMPE J.* **1982**, *18*, 19–24. |

| | | | | |
|---|---|---|---|---|
| -Ph-O-Ph- | Thermal polymerization in melt | Soluble | Linear polyene; in each link the group $-C_6H_4OC_6H_4C{\equiv}CH$ | [172] 172. Ratto, J.J.; et al. *J. Polym. Sci. Polym. Chem. Ed.* **1980**, *18*, 1035–1046, doi:10.1002/pol.1980.170180322. |
| -Ph-O-Ph- | $[(C_2H_5O)_3P]_4CoBr$ | Insoluble | Polycyclotrimer | [66] 66. Korshak, V.V.; et al.. US Patent US-3705131-A, filed 27.11.1970, and issued 1972. |
| -Ph-O-Ph- | $i\text{-}Bu_3Al\text{-}TiCl_4$ | Insoluble | Polycyclotrimer | [152] 15. Sergeyev, V.A.; et al. *Polymer Science U.S.S.R.* **1980**, *22*, 2130–2135, doi: 10.1016/0032-3950(80)90075-1. |
| $-Ph-Si(C_nH_{2n+1})_2-Ph-$ | $TaBr_5$ | Hyperbranched, soluble | The almost disappearance of groups –C≡CH | [173] 173. Liu, J.; et al. *Macromolecules* **2007**, *40*, 7473–7486, doi:10.1021/ma071062d. |
| $-Ph-CH_2-Ph-$ | Thermal polymerization | | Precursor of carbon/carbon composites | [12] 12. Bilow, N.; et al. *SAMPE J.* **1982**, *18*, 19–24. |
| -Ph-C(Ph)=C(Ph)-Ph-; $-PhO(CH_2)_6-OPh-$; $-CH_2OPh-CH_2-PhOCH_2-$; $-C(O)-PhO-(CH_2)_6-OPh-C(O)-$ | $InCl_3$/2-iodophenol | Regioregular hyperbranched soluble polymers | Absence of groups –C≡CH | [174] 174. Wang, Z.; et al. *Polym. Chem.* **2014**, *5*, 5890–5894, doi:10.1039/C4PY00859F. |

[a] information is taken from the primary sources indicated in the last column
